# Transactional Associations Between Bottle to Bed and Infant Sleep Problems Over the First Year

**DOI:** 10.1111/jsr.70237

**Published:** 2025-11-06

**Authors:** Esther M. Leerkes, Agona Lutolli, Cheryl Buehler, Lenka Shriver, Laurie Wideman

**Affiliations:** ^1^ UNC Greensboro Greensboro North Carolina USA

**Keywords:** bottle to bed, feeding practices, infant sleep problems, transactional model

## Abstract

The purpose of this study was to examine associations between putting the infant to bed with a bottle and maternal‐reported infant sleep problems using a 3‐wave cross‐lagged model. Participants included 299 mother‐infant dyads. When infants were 2, 6 and 14 months old, mothers reported their feeding practices using the Infant Feeding Practices Questionnaire II and infant sleep problems using the Brief Infant Sleep Questionnaire. Over and above covariates (maternal education, race, WIC participation, depressive symptoms, maternal sleep quality, breastfeeding status and weekly work hours), concurrent associations and stability pathways, putting the infant to bed with a bottle at 2 months predicted higher infant sleep onset latency, time awake at night and frequency of night wakings at 6 months. Infant nighttime sleep duration and frequency of night wakings at 6 months predicted greater maternal use of bottle to bed at 14 months. The indirect pathway from bottle to bed at 2 months to bottle to bed at 14 months via frequency of infant night wakings at 6 months was statistically significant supporting the transactional model whereby both mothers and infants influence the other's subsequent behaviour. The importance of preventing mothers from providing a bottle to bed and strategies to do so are discussed.

## 1 Introduction

Infant sleep problems convey risk for subsequent maladaptive child outcomes, including overweight and obesity (Alamian et al. 2016; Li et al. 2022; Tikotzky et al. 2010) and for negative parent and familial outcomes such as elevated depressive symptoms and less adaptive parenting and coparenting (Bai et al. 2020; McDaniel and Teti 2012). Notably, scholars have argued that parenting and infant sleep influence one another in a bidirectional and transactional manner, whereby infant sleep problems contribute to less adaptive parenting and less adaptive parenting contributes to worsening infant sleep problems over time (Burnham et al. 2002; Sadeh et al. 2010). However, empirical tests of transactional effects between parent behaviour and infant sleep outcomes using longitudinal cross‐lagged models are scant. One notable exception is a study by Fiese et al. (2021) in which parent provision of consistent bedtime routines predicted children's better sleep outcomes over time, and children's better sleep predicted more consistent bedtime routines. However, different child sleep components were implicated in the parent (nighttime waking and sleep problems) versus child‐driven paths (bedtime). In the current report, we employ cross‐lagged models to examine the extent to which providing a bottle to bed and four specific infant nighttime sleep components reflecting sleep problems are related over three time points (2, 6 and 14 months postpartum) in a diverse community sample. Notably, bottle to bed has been implicated as a risk factor for subsequent rapid infant weight gain and obesity, and as such is considered an obesogenic feeding practice (Appleton et al. 2018; Gibbs and Forste 2014; Wu et al. 2022). Thus, results have implications for both sleep health and physical health more broadly.

It is broadly believed that infants benefit from practice at soothing themselves to sleep at bedtime and when they awaken at night, essentially becoming more skilled at doing so independently (Anders and Keener 1985; Sadeh et al. 2010). Parental behaviours that undermine infant opportunities to self‐soothe to sleep are believed to contribute to poorer sleep outcomes such as longer sleep onset latency, more frequent and longer night wakings (i.e., sleep fragmentation) and less nighttime sleep overall. As such, expert guidance on promoting infant sleep health emphasises putting infants to bed while drowsy but alert as opposed to lulling them to sleep via feeding or other methods (Mindell et al. 2006; Paul et al. 2016). Consistent with this view, empirical evidence demonstrates that feeding infants to sleep is linked with their shorter nighttime sleep duration, more frequent night wakings overall and fewer night wakings in which they successfully self‐soothe back to sleep (Adams et al. 2022; Anuntaseree et al. 2008; Sadeh et al. 2009). Moreover, a randomised controlled intervention focused on training parents to put infants to bed while drowsy but awake, not to feed them to sleep and not to use food to soothe infants has been efficacious in promoting better infant sleep, providing the strongest empirical evidence of a potential causal link (Paul et al. 2016; Hohman et al. 2022).

Compromised infant sleep is believed to undermine adaptive parenting by reducing parental energy, regulatory capacity and attention and by increasing maternal negative mood and cognitions (Bai et al. 2020; McQuillan et al. 2019) and by making mothers less resilient in the face of parenting challenges (e.g., low social information processing skills) (Chary et al. 2020; King et al. 2020; Leerkes et al. 2024). Each of these studies provides empirical support for an association between infant sleep problems and less adaptive parenting outcomes such as lower parental sensitivity and emotional availability and higher negative parenting. The extent to which infant sleep predicts the use of bottle to bed specifically remains unknown. However, that maternal sleep problems, a correlate of infant sleep, predicted mothers' greater use of food to soothe when infants were 6 months old provides evidence of the effects of infant sleep on feeding practices generally (Leerkes et al. 2022). Given that providing a bottle at bedtime conveys risk for subsequent adverse weight outcomes, this is a significant gap in the literature. Such information could inform efforts to target and tailor behavioural interventions to be maximally efficacious, underscoring the importance of the current study.

The goal of the current study is to test the extent to which providing a bottle to bed and infant sleep problems predict one another over time, over and above stability paths and concurrent associations. We hypothesized that (a) mothers' greater use of putting the infant to bed with a bottle would predict more infant sleep problems over time, and (b) greater infant sleep problems would predict mothers' higher use of bottle to bed over time. Moreover, we hypothesized two possible indirect effects: (1) mothers' higher use of bottle to bed would predict elevated infant sleep problems over time which in turn would predict mothers' greater use of these obesogenic feeding practices over time; and (2) infant sleep problems would predict mothers' higher use of bottle to bed over time which in turn would predict infants' subsequent elevated sleep problems. If such indirect effects are statistically significant, they would provide the strongest evidence of transactional effects whereby mothers and infants shape one another's behaviour over time (Sameroff 2009).

Importantly, in the larger study from which these data were drawn, infant sleep was not a central focus. As such, objective measures of sleep like actigraphy were not collected. Although it is less than ideal that mothers reported on sleep and parenting given the potential for shared method variance, we control for maternal depressive symptoms at each wave to minimise negative response bias generally. In addition to controlling for stable factors known to influence maternal feeding practices (education, race/ethnicity, income, breastfeeding knowledge; Kalhor et al. 2025), we controlled for maternal sleep quality, weekly work hours and breastfeeding status at each wave to rule out competing explanations for observed effects. This includes possible effects of maternal fatigue driven by non‐infant factors, including employment on feeding practices (Hawkins et al. 2007) and feeding mode on infant night wakings (Galbally et al. 2013). We included participation in the Women, Infant and Children Special Food Supplemental Program (WIC) as a covariate to reflect both income as it is an income eligibility program and as a proxy for breastfeeding knowledge as participants are required to participate in breastfeeding education classes (USDA 2006).

## 2 Method

### 2.1 Participants

Pregnant women in their third trimester were recruited in and around Guilford County, North Carolina, to participate in the Infant Growth and Development Study (Leerkes et al. 2020). The primary aims of this study were to identify early life predictors of childhood obesity. Recruitment strategies included outreach through childbirth education classes, WIC breastfeeding classes, OB/GYN clinical flyers and social media platforms. To be eligible, participants had to be at least 18 years old, expecting a singleton, able to read and understand English, and planning to remain in the region for at least 3 years. Of the original 299 participants, most women (*n* = 269, *n* = 243 and *n* = 226) provided some data at the 2‐(90%), 6‐(81%) and/or 14‐(76%) month waves. Reasons for missing data included inability to contact the mother within the time frame, withdrawal from the study, or ineligibility due to infant mortality or health complications.

### 2.2 Procedure

During the third trimester of pregnancy (*M* gestational age = 33.75 weeks, SD = 3.52), expectant mothers provided electronic or written consent and completed online questionnaires, including demographics (e.g., educational attainment, race and income), using Qualtrics. Infant birth information (e.g., date of birth, sex, birthweight) was collected via phone interview approximately 1 week following the infant's due date. Mothers who remained eligible completed online questionnaires prior to each postpartum lab visit (*M* = 2.30 months, SD = 0.57; *M* = 6.90 months, SD = 1.18; *M* = 14.73 months, SD = 1.15) about their mental health, sleep quality, employment status, participation in WIC and feeding mode(s). In addition, at every wave mothers reported their infant's sleep and the frequency with which they used food to soothe their infant and put their infant to bed with a bottle. Participants received financial incentives for completing each wave as follows: $50 prenatal, $70 2 months, $80 6 months and $90 14 months. All procedures were approved by the University of North Carolina Greensboro Internal Review Board (#18‐0198). Artificial intelligence was not used in the design, implementation, analysis, or writing of this study/manuscript.

### 2.3 Measures

#### 2.3.1 Bottle to Bed

Mothers reported their frequency of putting their infant to bed with a bottle of formula, breast milk, juice, juice drink, or any other kind of milk using one item from the Infant Feeding Practices Questionnaire‐II (IFP; Fein et al. 2008) when their infants were 2, 6 and 14 months old. Mothers rated this on a scale of 1 (*at most bedtimes, including naps*) to 5 (*never*). This item was reverse coded so that higher scores indicated greater use of putting the infant to bed with a bottle.

#### 2.3.2 Sleep Problems

Mothers reported their infant's sleep problems at 2, 6 and 14 months using the Brief Infant Sleep Questionnaire, a widely used measure developed to assess sleep patterns of infants based on parents' report (Sadeh 2004). Four raw items served as separate indicators of infant sleep problems as follows: frequency of night wakings (“How many night wakings does infant have per night on average?” reported as a frequency count), nocturnal sleep onset latency (“How long does it take to put infant to sleep on average?” reported in minutes), nocturnal sleep duration (“How much time does your infant spend asleep during the night on average?” reported in hours) and duration of night wakefulness (“How much time during the night does your infant spend in wakefulness on average?” reported in minutes).

#### 2.3.3 Covariates

Maternal education, race and WIC participation were entered as time‐invariant covariates specified on the 2‐month variables. Mothers reported education, race, ethnicity, income, household composition, parity and partner status prenatally. Family income‐to‐needs ratio was calculated by dividing the total household income by the federal poverty threshold for that household size and year. A ratio of 1.0 indicates income at the poverty line; values below 1.0 reflect income below the poverty line, and values above 1.0 reflect income above the poverty line. *Maternal education* was coded on a 7‐point scale ranging from (1) some high school to (7) graduate degree. *Race* was coded as (0) Black, Multiracial, Hispanic and Other and (1) Non‐Hispanic White. Mothers reported on *WIC participation* on the IFP at each wave; participation was coded as 1 and not participating as 0. Participation in WIC was highly stable across waves (*r*s > 0.80, all *p* < 0.001), justifying the use of the 2‐month measure as a time‐invariant covariate.

In addition, maternal depressive symptoms, sleep quality, breastfeeding status and weekly work hours at each wave were specified as time‐varying covariates. *Depressive symptoms* were assessed using the 20‐item Center for Epidemiologic Studies‐Depression scale (CES‐D; Radloff 1977). Items were averaged; higher scores indicated more depressive symptoms (*α* > 0.89 for all waves). *Maternal sleep quality* was assessed via a modified version of the Pittsburgh Sleep Quality Inventory (Buysse et al. 1989), that included 4 of the 7 original sleep component scores: subjective sleep quality (1 item; how would you rate your sleep quality overall?), sleep duration (1 item; i.e., how many hours of actual sleep do you get a night?), sleep disturbances (9 items; e.g., how often in the past month did you have trouble sleeping because you have pain) and daytime dysfunction (1 item; i.e., how much of a problem has it been for you to keep up enough enthusiasm to get things done). Response scales varied across items and were thus standardised prior to calculating the mean value. Higher scores indicated poorer sleep quality (*α* > 0.67 for all waves). *Maternal breastfeeding status* was coded as (0) infant was not breastfed or fed breast milk in the past 7 days, and (1) infant was breastfed or fed breast milk in the past 7 days. Finally, mothers reported on the *number of hours they worked per week* on a continuous scale; mothers who were unemployed were coded as 0 h.

### 2.4 Analytic Approach

Preliminary and primary analyses were conducted using structural equation modelling (SEM) in Mplus Version 8.6 (Muthén and Muthén 2020). Full‐information maximum likelihood estimation was used to account for missing data. Preliminary analyses included estimating descriptives and intercorrelations for all variables. For the primary analyses, four cross‐lagged panel models were conducted to examine associations between bottle to bed and mother‐reported infant sleep problem components at infant ages 2, 6 and 14 months. Separate models were conducted for each sleep component (i.e., nighttime sleep duration, onset latency, time awake and frequency of wakings). This analytic model enables the simultaneous examination of three types of associations: (1) stability coefficients reflecting the consistency of repeated measures over time (e.g., bottle to bed at 2 months and bottle to bed at 6 months); (2) concurrent correlations between maternal feeding practices and mother‐reported infant sleep problems at each time point; and (3) unique cross‐lagged associations that capture the prospective effects of earlier maternal feeding practices on later infant sleep problems, and vice versa. The 2‐month dependent variables (i.e., bottle to bed and infant sleep problems) were regressed on time‐invariant covariates, including maternal education, race and income, drawn from the prenatal or 2‐month wave. Each dependent variable was estimated using time‐varying covariates measured at the same time point (i.e., 2, 6 and 14 months); these time‐varying covariates were maternal depressive symptoms, maternal sleep quality, breastfeeding status and hours worked. Indirect effects were evaluated using bootstrapped 95% confidence intervals, with statistical significance indicated by intervals that did not include 0.

## 3 Results

### 3.1 Sample Characteristics

Sample characteristics are displayed in Table 1. Mothers in the analytic sample had a mean age of 29.71 years (SD = 5.48) and an average income‐to‐needs ratio of 3.49 (SD = 2.96). The sample was racially and ethnically diverse, with 55.2% identifying as White, 29.4% as Black/African American and 7.7% identifying as Hispanic or Latino. Educational attainment varied, with 43.8% of mothers holding a four‐year college degree or higher; 18.1% had a high school diploma or less. Most mothers reported having a partner in the home during pregnancy (82.9%) and 41.8% were first‐time mothers. Approximately 39% of mothers participated in WIC when infants were 2 months old. Infants were evenly distributed across sex, with 49.5% being male.

**TABLE 1:** Sample characteristics.

Variable	*N*	*M* or %	SD
Maternal age	297	29.71	5.48
Family income to needs ratio	290	3.49	2.96
WIC participant at 2 months			
Yes	115	38.5	
No	135	45.2	
Not reported	49	16.4	
Maternal Race			
White	165	55.2%	
Black/African American	88	29.4%	
Other	15	5.0%	
Multiracial	20	6.7%	
Not reported	11	3.7%	
Maternal Ethnicity			
Hispanic or Latino	23	7.7%	
Not Hispanic or Latino	272	91.0%	
Not reported	4	1.3%	
Maternal Education Level			
High school diploma/GED or less	54	18.1%	
Some college	55	18.4%	
2‐year college degree	17	5.7%	
4‐year college degree	68	22.7%	
Post graduate work/degree	63	21.1%	
Not reported	42	14.0%	
Partner in the home (prenatal)			
Yes	248	82.9%	
No	47	15.7%	
Not reported	4	1.3%	
Parity status			
First time mother	125	41.8%	
Multiparous mothers	174	58.2%	
Infant Sex			
Female	141	47.2%	
Male	148	49.5%	
Not reported	10	3.3%	

### 3.2 Preliminary Analyses

Descriptive statistics and correlations among primary variables appear in Table 2. Three time‐invariant covariates (maternal education, race/ethnicity and income) and four time‐varying covariates measured at 2, 6 and 14 months (maternal depressive symptoms, maternal sleep quality, breastfeeding status and hours worked) were examined as potential covariates. Compared with other mothers, mothers who were non‐Hispanic White, had higher educational attainment and were not WIC participants at 2 months reported less frequent use of a bottle to bed at all waves and generally better quality infant sleep across components and waves.

**TABLE 2:** Descriptive statistics and correlations among all variables.

Variable	*M* (SD)	1	2	3	4	5	6	7	8	9	10	11	12	13	14
1. Maternal Education	4.62 (1.84)	—													
2. WIC Participant 2 months^a^	0.46 (NA)	−0.65**	—												
3. M Non‐Hispanic White^a^	0.53 (NA)	0.38**	−0.59**	—											
4. Depressive Symptoms 2 months	0.52 (0.43)	−0.20**	0.16**	−0.18**	—										
5. Depressive Symptoms 6 months	0.51 (0.45)	−0.19**	0.16**	−0.15**	0.53**	—									
6. Depressive Symptoms 14 months	0.51 (0.48)	−0.28**	0.29**	−0.22**	0.64**	0.65**	—								
7. M Poor Sleep Quality 2 months	1.34 (0.5)	−0.10	0.02	−0.12*	0.51**	0.28**	0.37**	—							
8. M Poor Sleep Quality 6 months	1.19 (0.55)	−0.09	0.06	−0.04	0.44**	0.56**	0.52**	0.48**	—						
9. M Poor Sleep Quality 14 months	1.15 (0.57)	−0.14*	0.16**	−0.09	0.43**	0.43**	0.68**	0.46**	0.65**	—					
10. Infant Breastfed 2 months^a^	0.78 (NA)	0.35**	−0.31**	0.22**	−0.16**	−0.10	−0.12*	−0.03	−0.02	−0.06	—				
11. Infant Breastfed 6 months^a^	0.61 (NA)	0.35**	−0.32**	0.29**	−0.24**	−0.11	−0.14*	−0.11	−0.04	−0.06	0.63**	—			
12. Infant Breastfed 14 months^a^	0.35 (NA)	0.14**	−0.14*	0.12*	−0.14*	−0.20**	−0.12*	0.02	−0.06	−0.06	0.35**	0.56**	—		
13. Work Hours 2 months	32.85 (13.19)	0.10	−0.22**	−0.00	−0.05	−0.07	−0.05	0.06	0.01	0.04	−0.05	−0.07	−0.11	—	
14. Work Hours 6 months	33.77 (14.22)	0.18**	−0.16**	−0.07	−0.04	−0.10	−0.09	0.07	0.05	0.04	0.05	−0.04	−0.09	0.57**	—
15. Work Hours 14 months	35.06 (11.39)	0.26**	−0.36**	0.09	0.04	−0.09	−0.02	0.05	−0.01	0.01	0.05	−0.06	−0.08	0.59**	0.59**
16. Bottle to Bed 2 months	2.88 (1.79)	−0.16**	0.25**	−0.27**	0.06	0.04	0.15*	−0.03	0.02	0.04	−0.19**	−0.23**	−0.15**	−0.19**	−0.04
17. Bottle to Bed 6 months	2.48 (1.68)	−0.18**	0.18**	−0.25**	0.07	0.09	0.09	−0.01	0.05	−0.05	−0.08	−0.15**	−0.16**	−0.04	−0.10
18. Bottle to Bed 14 months	2.71 (1.74)	−0.16**	0.16**	−0.30**	0.20**	0.20**	0.21**	0.04	0.16**	0.11	−0.12*	−0.05	−0.03	0.16**	−0.07
19. Sleep Duration 2 months	7.61 (2.17)	0.38**	−0.39**	0.42**	−0.19**	−0.14*	−0.22**	−0.34**	−0.11	−0.14*	0.19**	0.29**	0.13*	−0.02	−0.04
20. Sleep Duration 6 months	8.87 (2.09)	0.29**	−0.41**	0.45**	−0.05	−0.17**	−0.12*	−0.05	−0.15**	−0.10	0.21**	0.20**	0.17**	−0.01	−0.08
21. Sleep Duration 14 months	9.91 (1.77)	0.40**	−0.48**	0.57**	−0.08	−0.21**	−0.22**	−0.04	−0.09	−0.15**	0.20**	0.22**	0.12*	−0.11	−0.03
22. Sleep Onset Lat 2 months	40.80 (35.04)	−0.07	0.02	−0.06	0.27**	0.19**	0.22**	0.24**	0.26**	0.24**	−0.03	0.01	0.04	−0.03	0.11
23. Sleep Onset Lat 6 months	28.00 (21.09)	−0.22**	0.17**	−0.15**	0.27**	0.31**	0.25**	0.12*	0.28**	0.28**	−0.11	−0.10	−0.01	−0.13*	−0.13*
24. Sleep Onset Lat 14 months	22.20 (18.42)	−0.23**	0.28**	−0.33**	0.22**	0.22**	0.33**	0.22**	0.19**	0.32**	−0.14*	−0.15**	0.01	0.06	0.02
25. Time Awake Night 2 months	102.53 (77.51)	−0.16**	0.19**	−0.22**	0.18**	0.06	0.19**	0.23**	0.12*	0.16**	−0.10	−0.18**	−0.09	−0.02	0.02
26. Time Awake Night 6 months	55.76 (85.36)	−0.09	0.12*	−0.20**	0.08	0.15**	0.17**	0.09	0.18**	0.15**	−0.13*	−0.02	−0.01	−0.03	0.02
27. Time Awake Night 14 months	23.16 (36.34)	−0.24**	0.31**	−0.34**	0.09	0.22**	0.23**	0.02	0.04	0.19**	−0.19**	−0.17**	−0.04	0.05	−0.00
28. # Night Wakings 2 months	2.07 (1.05)	−0.04	0.07	−0.14*	0.12*	0.18**	0.18**	0.24**	0.27**	0.17**	−0.02	0.01	−0.03	0.08	0.04
29. # Night Wakings 6 months	1.56 (1.18)	−0.05	0.04	−0.06	0.06	0.15**	0.11	0.04	0.28**	0.15**	0.02	0.10	0.13*	−0.12*	−0.02
30. # Night Wakings 14 months	0.92 (1.07)	−0.11	0.18**	−0.23**	−0.02	0.11	0.13*	−0.06	0.18**	0.19**	0.05	0.12*	0.23**	−0.02	−0.01

Variable	15	16	17	18	19	20	21	22	23	24	25	26	27	28	29	30
1. Maternal Education																
2. WIC Participant 2 months^a^																
3. M Non‐Hispanic White																
4. Depressive Symptoms 2 months																
5. Depressive Symptoms 6 months																
6. Depressive Symptoms 14 months																
7. M Poor Sleep Quality 2 months																
8. M Poor Sleep Quality 6 months																
9. M Poor Sleep Quality 14 months																
10. Infant Breastfed 2 months^a^																
11. Infant Breastfed 6 months^a^																
12. Infant Breastfed 14 months^a^																
13. Work Hours 2 months																
14. Work Hours 6 months																
15. Work Hours 14 months	—															
16. Bottle to Bed 2 months	−0.03	—														
17. Bottle to Bed 6 months	−0.04	0.37**	—													
18. Bottle to Bed 14 months	−0.05	0.18**	0.36**	—												
19. Sleep Duration 2 months	0.06	−0.13*	−0.04	−0.20**	—											
20. Sleep Duration 6 months	−0.02	−0.19**	−0.17**	−0.26**	0.47**	—										
21. Sleep Duration 14 months	−0.03	−0.17**	−0.20**	−0.21**	0.45**	0.61**	—									
22. Sleep Onset Lat 2 months	0.03	−0.12*	−0.03	0.02	−0.15*	−0.03	0.03	—								
23. Sleep Onset Lat 6 months	−0.12*	0.12*	0.10	0.09	−0.16**	−0.29**	−0.24**	0.43**	—							
24. Sleep Onset Lat 14 months	0.03	0.12*	0.14*	0.19**	−0.31**	−0.22**	−0.39**	0.20**	0.44**	—						
25. Time Awake Night 2 months	−0.03	0.13*	0.16**	0.16**	−0.29**	−0.15*	−0.07	0.29**	0.18**	0.20**	—					
26. Time Awake Night 6 months	0.06	0.16**	0.09	0.13*	−0.20**	−0.33**	−0.21**	0.09	0.22**	0.15**	0.21**	—				
27. Time Awake Night 14 months	−0.02	0.08	0.04	0.20**	−0.21**	−0.29**	−0.43**	0.08	0.34**	0.41**	0.24**	0.26**	—			
28. # Night Wakings 2 months	−0.00	0.02	0.07	0.03	−0.13*	−0.02	−0.05	0.15**	0.04	0.01	0.33**	0.12*	0.04	—		
29. # Night Wakings 6 months	−0.12*	0.20**	0.16**	0.23**	−0.10	−0.24**	−0.17**	0.11*	0.31**	0.18**	0.14**	0.25**	0.20**	0.32**	—	
30. # Night Wakings 14 months	0.07	0.02	0.01	0.19**	−0.10	−0.23**	−0.33**	0.05	0.35**	0.30**	0.01	0.22**	0.39**	0.13*	0.54**	—

Abbreviations: Lat = latency; M = mother.

^a^ Given variable is dichotomous, values are Spearman correlations and percent is provided rather than mean.

* *p* < 0.05.

** *p* < 0.01.

Turning to the time‐varying covariates, we focus on concurrent associations with key variables as they are of primary interest. Elevated maternal depressive symptoms demonstrated significant concurrent associations with (a) maternal reports of poorer infant sleep across all components at all waves and (b) greater use of bottle to bed at 14 months. Poorer maternal sleep quality demonstrated significant concurrent associations with mother‐reported poorer infant sleep across all components at all 3 waves. Higher maternal work hours were concurrently significantly associated with (a) less use of bottle to bed at 2 months and (b) shorter mother‐reported infant sleep onset latency at 6 months. Breastfeeding was significantly concurrently associated with (a) less frequent provision of a bottle at bedtime at 2 and 6 months, (b) higher mother‐reported sleep duration at all waves, and (c) more frequent night wakings at 14 months only.

### 3.3 Primary Analyses

To test our hypotheses, we estimated four cross‐lagged panel models examining the bidirectional associations between mothers' use of bottle to bed and mother‐reported infant sleep components (i.e., 1 model for each of 4 infant sleep components). All models controlled for three time‐invariant covariates measured at 2 months (maternal education, race/ethnicity and WIC participation) and four time‐varying covariates assessed at each wave (maternal depressive symptoms, maternal poor sleep quality, breastfeeding status and work hours). The associations between time‐invariant covariates and the outcomes at 2 months, as well as the associations between time‐varying covariates and their corresponding wave‐specific outcomes, are presented in Table 3.

**TABLE 3:** Associations between covariates and outcome variables across waves.

Covariate (wave)	Outcome wave	Bottle to bed^a^	Sleep duration	Sleep onset lat	Time awake	# Night wakings
		*β* (*p*)	*β* (*p*)	*β* (*p*)	*β* (*p*)	*β* (*p*)
Time‐Invariant Covariates						
Maternal Education (p)	2 months	0.02 (0.79)	**0.18 (0.01)**	−0.05 (0.61)	−0.03 (0.74)	0.03 (0.74)
M Non‐Hispanic White (p)	2 months	**−0.22 (< 0.01)**	**0.24 (0.01)**	−0.03 (0.68)	−0.13 (0.14)	−0.12 (0.21)
WIC Participation (2 months)	2 months	0.06 (0.57)	−0.14 (0.19)	−0.06 (0.60)	0.08 (0.41)	0.04 (0.73)
Time‐Varying Covariates						
M Depressive Sym (2 months)	2 months	0.02 (0.82)	0.08 (0.25)	**0.20 (0.02)**	0.04 (0.63)	−0.01 (0.87)
M Poor Sleep Quality (2 months)	2 months	−0.05 (0.51)	**−0.32 (0.00)**	0.13 (0.10)	**0.19 (0.02)**	**0.23 (0.00)**
Infant Breastfed (2 months)	2 months	−0.13 (0.06)	0.04 (0.56)	0.01 (0.89)	−0.03 (0.68)	0.02 (0.78)
Work Hours (2 months)	2 months	−0.12 (0.28)	−0.04 (0.66)	−0.03 (0.81)	0.01 (0.93)	0.07 (0.64)
M Depressive Sym (6 months)	6 months	0.05 (0.44)	−0.07 (0.34)	**0.15 (0.04)**	0.08 (0.38)	−0.01 (0.92)
M Poor Sleep Quality (6 months)	6 months	−0.01 (0.93)	−0.05 (0.53)	0.09 (0.21)	0.11 (0.24)	**0.21 (0.01)**
Infant Breastfed (6 months)	6 months	−0.05 (0.27)	0.03 (0.67)	−0.06 (0.32)	0.05 (0.43)	**0.16 (0.01)**
Work Hours (6 months)	6 months	−0.07 (0.39)	−0.09 (0.23)	**−0.17 (0.05)**	0.06 (0.41)	−0.05 (0.53)
M Depressive Sym (14 months)	14 months	**0.17 (0.04)**	−0.16 (0.07)	0.14 (0.14)	0.15 (0.25)	0.03 (0.71)
M Poor Sleep Quality (14 months)	14 months	−0.00 (0.92)	0.00 (1.00)	0.14 (0.08)	0.08 (0.49)	0.11 (0.26)
Infant Breastfed (14 months)	14 months	0.04 (0.54)	−0.02 (0.74)	0.06 (0.27)	−0.01 (0.87)	**0.18 (0.01)**
Work Hours (14 months)	14 months	−0.04 (0.61)	−0.04 (0.56)	0.09 (0.18)	−0.03 (0.66)	0.11 (0.10)

*Note:* Bold values are statistically significant with *p* < 0.05. Abbreviations: lat = latency; M = mother; *p* = prenatal; sym = symptoms.

^a^ Values varied across models given other variables in the model varied, thus the mean value across models is presented for *β* and *p*.

**FIGURE 1:**
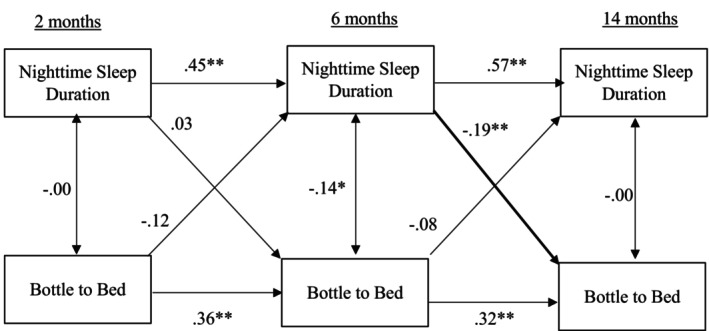
Cross lagged model for infant nighttime sleep duration and bottle to bed.

Standardised path coefficients for the cross‐lagged panel model examining bottle to bed and infant nighttime sleep duration are presented in Figure 1. All stability coefficients were positive and significant (bottle to bed: *β* = 0.36, *β* = 0.32, *p* < 0.01; infant sleep duration: *β* = 0.45, *β* = 0.57, *p* < 0.01, as reported chronologically). Of the concurrent associations, both bottle to bed and sleep duration were only significantly associated at 6 months, *β* = −0.12, *p* < 0.05. Infants who were put to bed with a bottle had shorter sleep duration. One significant cross‐lagged effect emerged: shorter sleep duration at 6 months predicted mothers' greater use of bottle to bed at 14 months, *β* = −0.19, *p* < 0.01.

**FIGURE 2:**
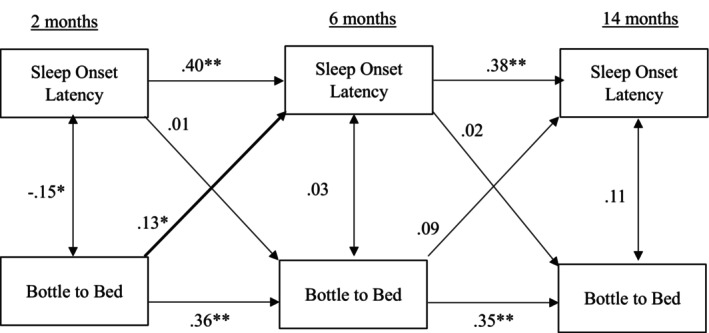
Cross‐lagged model for sleep onset latency and bottle to bed.

Results for the cross‐lagged panel model examining bottle to bed and sleep onset latency are presented in Figure 2. Stability coefficients were positive and significant (bottle to bed, not repeated as all comparable to model 1; infant sleep duration: *β* = 0.40, *β* = 0.38, *p* < 0.01, as reported chronologically). Of the concurrent associations, bottle to bed and sleep onset latency were only significantly associated at 2 months, *β* = −0.15, *p* < 0.05. Infants who were put to bed with a bottle had shorter onset latencies. One significant cross‐lagged effect emerged: bottle to bed at 2 months predicted longer sleep onset latency at 6 months, *β* = 0.13, *p* < 0.05.

**FIGURE 3:**
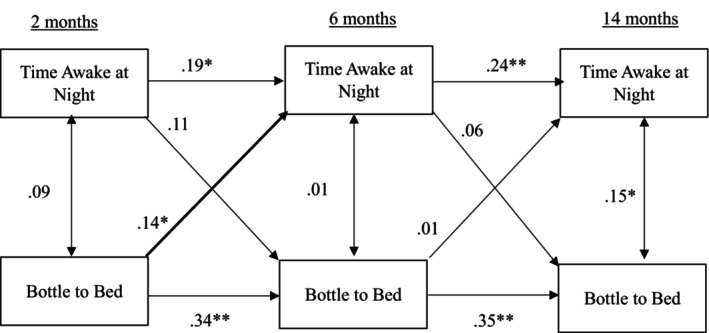
Cross‐lagged model for time awake at night and bottle to bed.

Results for the cross‐lagged panel model examining bottle to bed and time awake at night are presented in Figure 3. Stability coefficients were positive and significant (time awake at night: *β* = 0.19, *p* < 0.05; *β* = 0.24, *p* < 0.01, as reported chronologically). Of the concurrent associations, bottle to bed and time awake at night were only significantly associated at 14 months, *β* = 0.15, *p* < 0.05. Infants who were put to bed with a bottle spent more time awake at night. One significant cross‐lagged effect emerged: bottle to bed at 2 months predicted more time awake at 6 months, *β* = 0.14, *p* < 0.05.

**FIGURE 4:**
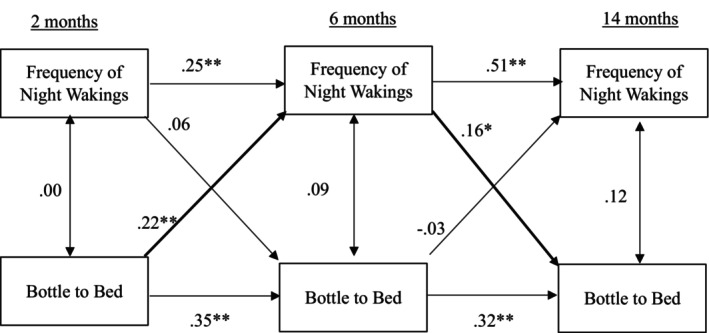
Cross‐lagged model for frequency of night wakings and bottle to bed.

Results for the cross‐lagged panel model examining bottle to bed and frequency of night wakings are presented in Figure 4. Stability coefficients were positive and significant (frequency of night wakings: *β* = 0.25, *β* = 0.51, *p* < 0.01, as reported chronologically). None of the concurrent associations between bottle to bed and frequency of night wakings were statistically significant. Two statistically significant cross‐lagged effects emerged: (a) bottle to bed at 2 months predicted more frequent night wakings at 6 months, *β* = 0.22, *p* < 0.01 and (b) more frequent night wakings at 6 months predicted more frequent use of bottle to bed at 14 months, *β* = 0.16, *p* < 0.05. To test the transactional nature of this relationship, we investigated the indirect effect of bottle to bed at 2 months on bottle to bed at 14 months via frequency of night wakings at 6 months. This indirect effect was statistically significant (*b* = 0.04, SE = 0.02, 95% CI [0.006, 0.082], *β* = 0.04) supporting a transactional pathway between bottle to bed feeding and more problematic infant sleep over time.

## 4 Discussion

The purpose of the current study was to examine the extent to which infant sleep problems and putting the infant to bed with a bottle predict one another across infancy. Results support both (a) mother‐driven effects whereby putting the infant to bed with a bottle at 2 months predicted mother‐reported longer sleep onset latency, greater time awake at night, and more frequent awakenings at nighttime at 6 months and (b) infant‐driven effects whereby both lower nighttime sleep duration and more frequent night wakings at 6 months predicted greater maternal use of bottle to bed at 14 months. These effects were over and above stability, concurrent associations and a host of covariates. Moreover, the entire indirect pathway from bottle to bed at 2 months to bottle to bed at 14 months via frequency of infant night wakings was statistically significant, providing strong evidence of a transactional effect. These findings support the view that lulling infants to sleep with a bottle undermines their sleep, perhaps by disrupting their ability to self‐soothe to sleep (Anders and Keener 1985; Sadeh et al. 2010), and that poor infant sleep disrupts adaptive parenting at bedtime (Fiese et al. 2021). To our knowledge, this is the first study to demonstrate the effect of infant sleep problems on parents' subsequent use of bottle to bed. Results also support the argument that the association between parenting and infant sleep is transactional in nature (Burnham et al. 2002; Sadeh et al. 2010).

Notably, three mother‐driven effects emerged from 2 to 6 months, whereas two infant‐driven effects emerged from 6 to 14 months. It may be that given low expectations for infant sleep in the perinatal period, mothers are able to cope more effectively with sleep problems without engaging in obesogenic nighttime feeding practices. However, when infant sleep problems persist over time, mothers' ability to cope and maintain recommended bedtime practices may wane. This points to the need for ongoing efforts to check in with mothers about infant sleep and to provide useful information and support at well‐baby visits and in other settings.

Only three out of 12 possible concurrent associations between mother‐reported maternal behaviour and infant sleep problems were significantly associated, which is contrary to some prior research (Sadeh et al. 2009). As would be expected, each behaviour of interest demonstrates significant stability over time, but stability was primarily in the moderate effect size range. This may be a function of normative maturation in infant sleep (e.g., increasing sleep consolidation) and eating patterns (e.g., capacity to eat food and drink from a cup, greater consumption of solids relative to liquids over time) and reduced use of breastfeeding over this period that contribute to change in nighttime sleep and bottle use for many infants and mothers. Given bottle to bed was a salient predictor of infant sleep problems, additional research to identify factors that predict initial and changing use of this parenting practice over time would be useful to inform targeted prevention and intervention programs.

The current study underscores the value of anticipatory guidance for parents advising them not to put their infants to bed with a bottle and supports the continued use of interventions that emphasise this while providing parents with concrete strategies. One such example is the Intervention Nurses Start Infants Growing on Healthy Trajectories (INSIGHT) intervention which emphasises, among other things, putting infants to bed when awake but sleepy, not feeding infants to sleep and not using food to comfort fussy infants. Results of randomised control trials demonstrate that participants were less likely to use a bottle to bed than control mothers (Savage et al. 2018), and their infants demonstrated better sleep, both those in the intervention and their siblings who were born after the intervention (Paul et al. 2016; Hohman et al. 2022). Although these messages are routine parts of anticipatory guidance for parents, they may not be sufficient. INSIGHT provides resources related to effective soothing techniques and bedtime routines that may better equip parents to prevent sleep problems and respond more adaptively when they occur. Anticipatory guidance could be enhanced by (a) explicitly noting not to feed the infant to sleep via the breast or bottle in addition to not placing them in the crib with a bottle and (b) providing suggestions for age‐appropriate bedtime routines that may promote drowsiness to serve as an alternative to feeding the infant to sleep. Further, such information should be provided in varied modes (e.g., written handouts, video demonstrations, discussion with parents about what is and is not working with their infant) to enrich the messaging and appeal to a broader audience of parents. Given greater evidence of child effects from 6 to 14 months than from 2 to 6 months, it is important that practitioners be reminded to provide information and support about bedtime practices throughout infancy and not just in the neonatal period.

Importantly, the extent to which parents use food to soothe their infants to sleep initially and then back to sleep at night is another viable mechanism, in addition to bottle to bed, which could disrupt infants' ability to self‐settle back to sleep (Adams et al. 2019; Touchette et al. 2005). However, the most frequently used measure of using food to soothe during infancy focuses on daytime, not nighttime use of the behaviour (Stifter and Moding 2015). As such, revising existing measures to capture the frequency with which parents feed their infants to sleep and upon waking at night generally and even if they think the infant is not hungry at the time, could be useful. These distinctions are important as some nighttime feeding, particularly in early infancy, is important to meet infants' nutritional needs so care must be taken not to discourage appropriate nighttime feeding.

Strengths of the current study include the use of a prospective longitudinal design allowing for a 3‐wave cross‐lagged panel, the community sample which was diverse with respect to race and socioeconomic status, and the careful consideration of multiple covariates. Weaknesses include shared method variance given all measures were maternal report. This is particularly problematic for sleep given evidence that maternal reports of sleep become less accurate when compared to actigraphy after 6 months (Tikotzky and Volkovich 2019). On the other hand, mothers' perceptions of infant sleep are likely what guide parenting decisions and as a result are of value for scholars and clinicians who wish to prevent maladaptive parenting practices.

In conclusion, putting infants to bed with a bottle and infant sleep problems influence one another across infants' first year and into their second year. Given infant sleep problems are a predictor of maladaptive infant (Chaput et al. 2017), parent (Bai et al. 2020) and family outcomes (McDaniel and Teti 2012), efforts to prevent parental use of this strategy are important to promote infant and parent well‐being.

## Author Contributions

E.M.L., C.B., L.S., and L.W. designed and secured funding for the larger study from which the data were derived. E.M.L. conceptualized the research questions addressed in this manuscript. A.L. conducted the analyses. E.M.L. and A.L. drafted and revised the manuscript. C.B., L.S., and L.W. edited the manuscript.

## Ethics Statement

The protocol was approved by UNC Greensboro's Internal Review Board (protocol #18‐0198). Participants provided electronic or written informed consent.

## Conflicts of Interest

The authors declare no conflicts of interest.

## Data Availability

The data that support the findings of this study are openly available in Harvard Dataverse at https://dataverse.harvard.edu/, reference number https://doi.org/10.7910/DVN/ME8BHO.
